# An algorithm for score aggregation over causal biological networks based on random walk sampling

**DOI:** 10.1186/1756-0500-7-516

**Published:** 2014-08-11

**Authors:** Dmitry M Vasilyev, Ty M Thomson, Brian P Frushour, Florian Martin, Alain Sewer

**Affiliations:** Selventa, One Alewife Center, Cambridge, MA 02140 USA; Philip Morris International R&D, Philip Morris Products S.A, Quai Jeanrenaud 5, 2000 Neuchâtel, Switzerland

**Keywords:** Causal biological network, Causal consistency, Signed graph, Spanning tree, Random walk

## Abstract

**Background:**

We recently published in *BMC Systems Biology* an approach for calculating the perturbation amplitudes of causal network models by integrating gene differential expression data. This approach relies on the process of score aggregation, which combines the perturbations at the level of the individual network nodes into a global measure that quantifies the perturbation of the network as a whole. Such "bottom-up" aggregation relates the changes in molecular entities measured by omics technologies to systems-level phenotypes. However, the aggregation method we used is limited to a specific class of causal network models called "causally consistent", which is equivalent to the notion of balance of a signed graph used in graph theory. As a consequence of this limitation, our aggregation method cannot be used in the many relevant cases involving "causally inconsistent" network models such as those containing negative feedbacks.

**Findings:**

In this note, we propose an algorithm called "sampling of spanning trees" (SST) that extends our published aggregation method to causally inconsistent network models by replacing the signed relationships between the network nodes by an appropriate continuous measure. The SST algorithm is based on spanning trees, which are a particular class of subgraphs used in graph theory, and on a sampling procedure leveraging the properties of specific random walks on the graph. This algorithm is applied to several cases of biological interest.

**Conclusions:**

The SST algorithm provides a practical means of aggregating nodal values over causally inconsistent network models based on solid mathematical foundations. We showed its utility in systems biology, where the nodal values can be perturbation amplitudes of protein activities or gene differential expressions, while the networks can be models of cellular signaling or expression regulation. Since the SST algorithm is based on general graph-theoretical considerations, it is scalable to arbitrary graph sizes and can potentially be used for performing quantitative analyses in any context involving signed graphs.

**Electronic supplementary material:**

The online version of this article (doi:10.1186/1756-0500-7-516) contains supplementary material, which is available to authorized users.

## Background

Recently, we developed an approach aimed at assessing network perturbation amplitudes (NPA) by integrating gene differential expression measured by transcriptomics with causal biological network models built from prior literature-derived knowledge [1]. Like other methods, our computational approach relies on the aggregation of scores defined on the network nodes [2]. This need for aggregating nodal values is essential for "bottom-up" reasoning in molecular systems biology and appeared mainly as the consequence of two factors. First, the advances in experimental omics expression profiling technologies have enabled the quantification of the behavior of thousands of molecular entities contained in the system, such as the more than 20,000 mRNA transcripts. Second, accumulated evidence shows that the biological processes underlying system-level functions are best understood in terms of the interactions between the individual molecular entities within pathways or networks [3]. This evidence has motivated large-scale mining of the relationships between the molecular entities reported in the literature to assemble knowledge-based pathways and networks [4]. Such assemblies enable the study of cell-, tissue-, or organ-level phenomena under "bottom-up" perspectives, such as the response to exposure to biologically active substances relevant to pharmacology and toxicology [5], which was one of the motivations for the present study. Additional details about the systems biology concepts used in this note are given in the Appendix.

The aggregation method involved in our NPA calculations uses the edges connecting nodes in a causal biological network model to combine the real-valued nodal scores. While the NPA assessment framework provides specific values for the nodal scores (see the Appendix for more details), the aggregation method in itself is applicable to any means of assigning real-valued scores or measurements to the nodes of a causal network model. It is designed to be in accordance with the information contained in a causal network model, which includes in particular signed relationships for every edge: +1 = ***A*** → ***B*** = "increase (decrease) in node ***A*** causes increase (decrease) in node ***B***", and -1 = ***A***—|***B*** = "increase (decrease) in node causes decrease (increase) in node ***B***". Our aggregation method works for the major class of network models termed "causally consistent", where the relative sign in {-1,+1} between any node pair {***A,B***} of the network can be unambiguously determined using the product of the edge signs along any path relating nodes ***A*** and ***B***. However, it cannot be applied to "causally inconsistent" network models, where the relative sign in {-1,+1} between some node pairs {***A,B***} of the network cannot be unambiguously determined and depends on the specific paths relating nodes ***A*** and ***B***. This limitation prevents the NPA approach from being applied to richer causal network models and thereby cover further relevant biological processes such as the cell cycle [6].

**Figure 1:**
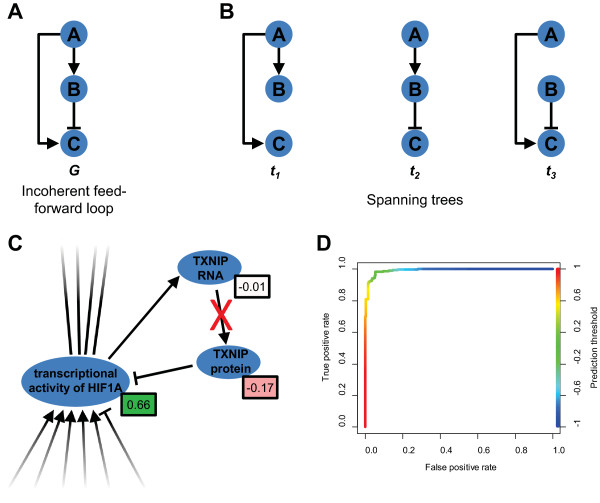
**Causally inconsistent biological networks, spanning trees, and results of the SST algorithm. (A)** The incoherent feed-forward loop (IFFL) as an example of a causally inconsistent network, termed an "unbalanced graph" in graph theory. **(B)** The three spanning trees corresponding to the IFFL shown in **(A)**. **(C)** Magnification of neighborhood of the TXNIP feedback loop from the "Hypoxic Stress" network. The effective node weights *S*_*n* → *REF*_ from SST are indicated in the boxes, and the red X indicates the edge that is absent in the pruned causally consistent version of the network. **(D)** Receiver operating characteristic (ROC) curve (true positive rate vs. false positive rate) for the comparisons between the effective node weights *S*_*n* → *REF*_ from SST and the corresponding nodal signs *s*_*n* → *REF*_ for the 19 networks given in Additional file 1: Table S1. The color of the curve follows the prediction threshold applied on *S*_*n* → *REF*_ and shows that mislabeling occurs mainly for small values around zero (i.e. the green part of the curve). The area under the ROC curve (AUROC) is 0.992.

To concretely illustrate how the causal inconsistency affects the possibility of aggregating real-valued score over a causal network model, we consider the incoherent feed-forward loop (IFFL) network motif shown in Figure 1A [7], which constitutes a simple but relevant case of a causally inconsistent network. The aggregation method used in the context of NPA calculations consists of summing the real-valued contributions of each node of the network, adjusted by their signs in {-1,+1} relative to one specific node called the "reference node" (see the Appendix for more details). The sign of a given node relative to the reference node is determined by the product of the edge signs along any (non-oriented) path relating that node and the reference node [1]. As mentioned above, such an approach is only possible when the network is causally consistent, that is when the relative sign in {-1,+1} between any node pair {***A*,*B***} can be unambiguously determined because it does not depend on the specific paths relating nodes ***A*** and ***B***. In the case of the causally inconsistent IFFL shown in Figure 1A, we observe that the two (non-oriented) paths "***A*** → ***B***" and "***A*** → ***C*** |— ***B*** " relating nodes ***A*** and ***B*** do not have the same sign, as obtained by the product of the signs of their edges. If node ***A*** is the reference node, then the relative sign of node ***A*** with respect to the reference node cannot be unambiguously determined. The same holds for the node pair (***A***, ***C***) and the corresponding paths "***A*** → ***C***" and "***A*** → ***B*** —| ***C***". These observations concretely exemplify the impossibility of performing the aggregation method necessary for NPA calculations when considering causally inconsistent network models.

A closer look into the aggregation methods used in the similar context of pathway analysis for the interpretation of differentially expressed genes revealed that none of the existing approaches were suitable for our task [2]. The main reason for this inadequacy is illustrated in the case of the "Signaling Pathway Impact Analysis" (SPIA) approach [8]: the information about pathway structure is used for node-level scoring and not for pathway-level aggregation, which is then not necessarily performed in full accordance with the pathway structure. The "shortest-path" approach constitutes another direction that was recently used to deal with causally inconsistent biological networks [9]. However, it is not satisfactory from the biological point-of-view, because a potentially relevant part of the biological information contained the network model is disregarded, as it can be clearly seen in the IFFL case. As a consequence, a novel approach is required for our task of aggregating real-valued scores over causally inconsistent networks.

In this note, we propose a novel algorithm called "sampling spanning trees" (SST) that extends our aggregation method to the class of "causally inconsistent" network models. This extension enables the NPA approach to be applied to richer causal network models and thereby cover further relevant biological processes such as the cell cycle [6]. In the "Findings" section, we present the mathematical concepts and properties underlying the SST algorithm, such as the notion of unbalanced graphs, which corresponds to causally inconsistent networks (see the Appendix for more details). We then show its application to several concrete cases, including the IFFL network motif and the calculations of perturbation amplitudes for causally inconsistent network models [6, 10–12]. We also explain the implementation of the SST algorithm in the case of these network models encoded in the Biological Expression Language (BEL) [13]. These considerations are accompanied by a comparison of the SST results with results obtained on quasi-equivalent causally consistent networks for which the SST algorithm was not necessary. The very good agreement between the two approaches confirms the appropriateness of our implementation. Overall, we find that the aggregation of nodal scores using the SST algorithm is mathematically solid and that its application to BEL-encoded causal network models is biologically sound. The SST algorithm can be applied to arbitrary-sized networks, and has potential utility beyond the case of causal network models used in systems biology, namely any situation where global quantities need to be calculated by aggregating the nodal values of an unbalanced graph consistently with the signs of its edges.

## Findings

### Overview

In this note, we propose a novel approach for unambiguously determining signed relationships between the nodes of a causally inconsistent network, which enables the application of the aggregation method used in our previous study [1]. This approach consists of replacing the "rigid" signs in {-1,+1} between the reference node and any other node by a "relaxed" continuous measure in [-1,+1] that characterizes relationships across the network (see the section "The SST algorithm" below). The value of this measure is calculated by averaging over a representative sample of spanning trees [14], which are suitably extracted from the causally inconsistent network, hence the algorithm name SST. Spanning trees represent meaningful quantities in the present context, because they constitute well-defined mathematical objects for encoding all the unambiguous pairwise relationships in {-1,+1} existing in a causally consistent network model. From that perspective, a causally inconsistent network model can be viewed as an "excessively rich" causal network model for which not all pairwise nodal relationships can be unambiguously determined, preventing the original aggregation method to be applied. However, by averaging over a representative sample of causally consistent spanning trees extracted from it, we ensure that the biological information used during the SST-based aggregation method is maximal and in full accordance with the network structure, so that the biological content of the network remains globally conserved.

### The SST algorithm

Given a connected balanced signed graph *G* = (Nodes, Edges) and a real-valued quantity *X*_*n*_ defined on the nodes of *G* (e.g., perturbation amplitude score [1] or gene differential expression [8]), we define the aggregation of *X*_*n*_ over *G* as
1

where *s*_*n* → *REF*_(*G*) ∈ {-1, 1} is the nodal sign given by the product of the edge signs over any path in *G* relating node *n* and the reference node *REF*. Since *G* is a balanced graph, *s*_*n* → *REF*_(*G*) is independent of the chosen path and is therefore defined unambiguously for all nodes *n*.

The SST algorithm is based on the concept of spanning trees: *t* is a spanning tree of *G* if *t* is a subgraph of *G* that is a tree and that connects all nodes of *G* [14]. The usefulness of spanning trees comes from the fact that the aggregated quantity *X*_*G*_ defined in Eq. (1) can be equally rewritten in terms of any spanning tree *t* of *G* as
2

where *s*_*n* → *REF*_(*t*) ∈ {-1, 1} is now calculated over the only path in *t* ⊂ *G* between node *n* and the reference node *REF*. Supposing that all the *N*(*G*) spanning trees *t*_1_, …, *t*_*N*(*G*)_ of *G* can be enumerated, *X*_*G*_ can be equivalently rewritten as
3

The key point of the SST algorithm is to realize that Eq. (3) is well-defined, even if *G* is an unbalanced graph. This property results from the fact that the enumeration of the spanning trees is independent of the edge signs (i.e. "→" or "—|") and from the fact that *s*_*n* → *REF*_(*t*) is unambiguously defined for a given spanning tree *t*. Swapping the summations over spanning trees *t* and nodes *n* in Eq. (3) yields the final expression
4

where
5

Eq. (4) extends the initial definition of *X*_*G*_, which is valid for balanced graphs only. It replaces the nodal sign *s*_*n* → *REF*_(*G*) ∈ {-1, + 1} by the nodal effective weights *S*_*n* → *REF*_(*G*) ∈ [-1, + 1] in the case of unbalanced graphs, representing a "structural average" over all possible spanning trees for which a well-defined aggregation of *X*_*n*_ over *G* can be calculated. The nodal effective weights *S*_*n* → *REF*_(*G*) also generalize beyond the specific aggregation method here (Eq. (1)), and represent a generally applicable "structurally averaged" signed relationship between two nodes (*n* and *REF*) in an unbalanced graph.

In practice, explicit enumeration of all the spanning trees is unrealistic for large unbalanced graphs. The second key element of the SST algorithm consists therefore in replacing the exhaustive sum over all spanning trees *t*_1_, …, *t*_*N*(*G*)_ in Eq. (5) by an approximation involving a computable representative subset of spanning trees *T*(*G*). We employed Aldous’ method that generates a suitable uniform sample of spanning trees using random walks over the graph [15]. This method consists of moving "signed" walkers along the graph, whose trajectory and sign in {-1,+1} are determined by the following set of local rules (assuming that *G* is connected):

1. Each walker starts at the reference node *REF*, with positive sign "+1".
2. The walker randomly chooses an edge connected to the current node to traverse. The edge choice is irrespective of the sign or direction of the edge.
3. The walker’s sign is preserved if it traverses a positively signed edge (+1 = "→") and is flipped if it traverses a negatively signed edge (-1 = "—|").
4. If the next node has not already been visited by that walker, the walker marks the next node as visited and the walker’s sign is assigned to that node.
5. If the next node has already been visited by that walker, then the walker adopts the sign from the node.
6. Continue until all nodes of the graph are visited.

In the framework of Aldous’ method explained above, Eq. (5) can be replaced by
6

where *N*_±_(*n*, *G*) and *N*_-_(*n*, *G*) record the number of random walkers visiting node *n* with positive and negative sign, respectively. *N*_+_(*n*, *G*) + *N*_-_(*n*, *G*) is the total number of sampled spanning trees in *T*(*G*), which is chosen to ensure convergence of the *S*_*n* → *REF*_(*G*) approximations. Note that the sampled spanning trees *t* ∈ *T*(*G*) are not needed explicitly for computing *S*_*n* → *REF*_(*G*) in Eq. (6). They can however be reconstructed for a given walker by collecting all the edges traversed during Step 4.

### Concrete applications of the SST algorithm

For illustration purposes, we first apply the SST algorithm to the IFFL mentioned in the "Background" section (Figure 1A). Since the three corresponding spanning trees {*t*_1_, *t*_2_, *t*_3_} are easily constructed (Figure 1B), we do not need the sampling part of the SST algorithm. Taking node *A* as the reference node *REF*, Eq. (5) yields directly the following aggregation weights: (*S*_*A* → *REF* = *A*_, *S*_*B* → *REF* = *A*_, *S*_*C* → *REF* = *A*_) = (1, 0.333, 0.333). Supposing the nodal values *X*_*A*_ = *X*_*B*_ = *X*_*C*_ = 1, which correspond to typical gene differential expressions, Eq. (4) gives an aggregated value *X*_*IFFL*_ = 1.667. This result is smaller than the sum of the individual values *X*_*A*_ + *X*_*B*_ + *X*_*C*_ = 3, reflecting the fact that these node values *X*_*n*_ are not consistent with the edge *B* —| *C* of the graph. In terms of spanning trees, Eq. (3) yields (*X*(*t*_1_), *X*(*t*_2_), *X*(*t*_3_)) = (3, 1, 1). This shows that the spanning tree *t*_1_ provides the highest contribution to *X*_*IFFL*_, which is due to it not containing the discrepant edge *B* —| *C*. Using other node values *X*_*n*_ enables us to make similar considerations. We conclude from this simple example that aggregation based on spanning trees gives quantitatively consistent results and that the results of both the node-based and the spanning tree-based representations can be meaningfully interpreted.

We then applied the SST algorithm to more complex causally inconsistent network models (including "Hypoxic Stress") that have been constructed to faithfully describe real biological processes in the lung, and therefore include negative feedback and contradictory regulatory relationships [6, 10–12]. Because of the particular semantics of the BEL language used to encode these network models, it was first necessary to slightly adapt the random walk rules to account for extra granularity in the network edges. This resulted in an implementation of the SST algorithm that contains an adequately modified Step 2, as explained in the Appendix.

To evaluate the results of the SST algorithm, we derived wherever possible pruned causally consistent versions of the causally inconsistent network models. Indeed, for many of these causally inconsistent networks we were able to manually remove a small number of edges to obtain reduced causally consistent networks that are biologically closest to the original networks. The pruned network models contain the same nodes as the original network models but differ by the signs of the relationships between some of their nodes. As such, they do not describe all the biological processes contained in the original network models and therefore should be applied preferably in the situations where the discarded processes do not constitute the dominant biology. If such situations are relevant in the cases where the network models will be considered, then using the pruned network models is justified (see the application to the TNF treatment dataset below for a concrete case). The decision to remove an edge was made based on the expected or desired causal relationships between each node and the reference node. For example, feedback loops were edited so that negative regulators were negatively related to the network through inhibitor activity, rather than positively related to the network through their transcriptional regulation. In so doing, we were able to compare the SST results calculated on the causally inconsistent networks (i.e., the effective nodal weights *S*_*n* → *REF*_(*G*) ∈ [-1, + 1]) with the aggregation results obtained on the corresponding pruned causally consistent network versions (i.e., the nodal signs *s*_*n* → *REF*_(*G*_PRUNED_) ∈ {-1, + 1}). As the SST algorithm essentially consists of combining many causally consistent versions of the original network model deduced from the sampled spanning trees, these comparisons provide valuable "biological benchmarks" for our results.

We first ran the SST algorithm on the "Hypoxic Stress" network model that contains 144 nodes and 241 edges [10]. Using 1,000 spanning trees was sufficient to produce nodal weights *S*_*n* → *REF*_(*G*_HS_) (as given by Eq. (6)) with a median difference of less than 0.01 from the nodal weights using 20,000 spanning trees (maximum difference less than 0.05). Additionally, a manual biological investigation was performed to produce a pruned causally consistent version of the network model that preserves its biological integrity by removing four edges (Additional file 1: Table S1). A comparison between the effective nodal weights *S*_*n* → *REF*_(*G*_HS_) from SST and the unambiguous nodal signs *s*_*n* → *REF*_(*G*_HS, PRUNED_) identified only a single node that differed in sign. A closer examination of the SST results revealed an interesting configuration in the region of the network magnified in Figure 1C: a causal inconsistency is present between the transcriptional activity of HIF1A (Hypoxia-inducible factor 1-alpha), the abundance of TXNIP (Thioredoxin-interacting protein) RNA, and the abundance of TXNIP protein. This causal inconsistency is indicated by the two paths "TXNIP protein —| HIF1A transcriptional activity" and "TXNIP protein ←TXNIP RNA ← HIF1A transcriptional activity" having opposite signs. The SST results indicate that the first path is preferred in the context of the aggregation over the "Hypoxic Stress" network: the "TXNIP protein" node has a negative sign and the "HIF1A transcriptional activity" node has a positive sign, in agreement with their negatively-signed connecting edge ("—|"). Furthermore, the effective weight from SST for the "TXNIP RNA" node is very close to zero, meaning that the sign of this node is largely ambiguous with respect to the reference node and thus this node has little contribution to aggregation over the "Hypoxic Stress" network. From a biological point of view, the edge connecting the "TXNIP RNA" node to the protein node was chosen to be removed (Additional file 1: Table S1), because the protein abundance and the activity of TXNIP are negative regulators of the pathway, and thus should have a negative contribution to the aggregated network score. These considerations are compatible with the SST results. This particular case indicates that the SST algorithm is scalable to more complex networks and that its results reflect the biological content of the network when considered in the aggregation context.

We further evaluated the results of the SST algorithm by assessing its performance against a set of graphs that were manually pruned to become causally consistent, as with the "Hypoxic Stress" network discussed above. Of the 81 (=15 + 7 + 32 + 23) networks contained in the cell proliferation, cellular stress, DNA damage/autophagy/cell death/senescence, and pulmonary inflammation publications [6, 10–12], 26 (=7 + 5 + 2 + 12) were causally inconsistent and 19 (=4 + 2 + 2 + 11) could be manually transformed into causally consistent networks using the same criteria as described above (Additional file 1: Table S1). We used the SST algorithm to compute the effective nodal weights *S*_*n* → *REF*_ ∈ [-1, + 1], and compared them with the nodal signs *s*_*n* → *REF*_ ∈ {-1, + 1} defined on the corresponding manually resolved networks. From the perspective of a classification problem, where *S*_*n* → *REF*_ give the predictions and *s*_*n* → *REF*_ the actual values, the SST algorithm exhibits high accuracy, with a 4.4% rate of mislabeling directions (using zero threshold for the *S*_*n* → *REF*_ values, and averaging across all networks; the mislabeling rate ranged from 0% to 19% for individual networks). The overall area under the receiver operating characteristic curve (AUROC) measured for the SST algorithm was 0.992 (ranging from 0.90 to 1.0 for individual networks), and the majority of mislabeling events occurred with effective nodal weights near zero (Figure 1D). These results show that the conclusions drawn for the SST algorithm in the case of the "Hypoxic Stress" network can be extended to other network models, which further supports the reliability of the approach.

**Figure 2:**
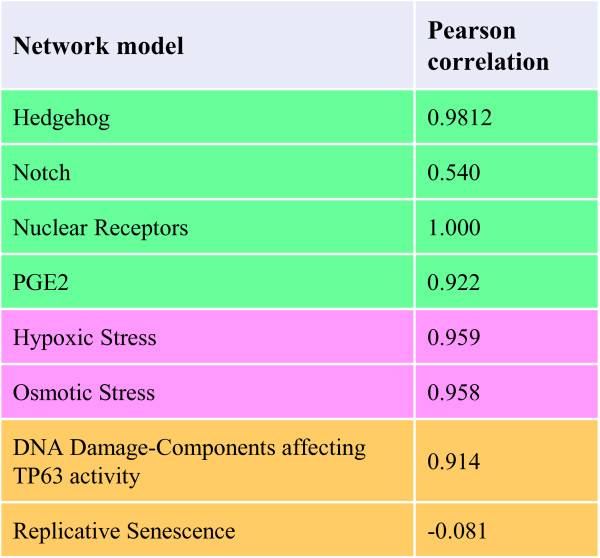
**Evaluation of the SST algorithm at the level of the NPA scores.** The Pearson correlation coefficients were calculated between the 16 pairs of GPI NPA scores corresponding to the causally inconsistent and pruned causally consistent network versions obtained for the 16 treatment vs. control comparisons contained in the TNF dataset. Only eight network models that were compatible with the tissue context of NHBE cells were considered. The low score correlation for the "Notch" network is consistent with the lack of significant scores for this network, while the poor score correlation for "Replicative Senescence" can be understood in light of the different effective nodal weights for nodes in a region of the network describing MAPK signaling.

Finally, we applied the SST algorithm to a concrete example of NPA and Biological Impact Factor (BIF) calculations requiring input gene differential expression data [1, 5, 16]. Note that because the SST algorithm is exclusively based on the prior biological knowledge of a network model, involving gene expression data will not change its actual results (i.e. the effective nodal weights *S*_*n* → *REF*_(*G*)). Rather, it will offer an additional perspective by reframing the *a priori* comparisons between the effective nodal weights *S*_*n* → *REF*_(*G*) and the nodal signs *s*_*n* → *REF*_(*G*_PRUNED_) in context of the *a posteriori* comparisons between the corresponding of NPA scores. We used a public data set describing the effect of TNF treatment of normal human bronchial epithelial (NHBE) cells, which was already used in our scoring framework [1, 17] (see the Appendix for more details). Of the 19 networks from Additional file 1: Table S1, the tissue contexts of eight networks were consistent with NHBE cells. The NPA GPI (geometric perturbation index) scores of these eight networks were computed using the effective nodal weights *S*_*n* → *REF*_ from SST for their causally inconsistent version and the nodal signs *s*_*n* → *REF*_ for their pruned causally consistent version [1, 16]. For each network, the scores were calculated for the 16 possible treatment versus control comparisons (four non-zero treatment doses and four time points). The results are displayed in Figure 2. Six of the eight networks displayed correlations of about 0.9. The poor correlation for the "Notch" network is consistent with the fact that none of the scores passed the Specificity and Uncertainty criteria, indicating that these scores largely reflect noise rather than true biological perturbation [1]. The poor correlation for the "Replicative Senescence" network results from the fact that all of the nodes with different signs for *S*_*n* → *REF*_ and *s*_*n* → *REF*_ lie in a single causally inconsistent region of the network that relates the impact of replicative senescence to MAPK signaling. Consistent with known TNF activation of MAPKs [18], this region of the network is rich in nodes with significant scores (based on Specificity and Uncertainty criteria [1]), often containing as many nodes with significant scores as the rest of the network despite being comprised of only ~20% of all nodes. This important finding illustrates that while the SST algorithm is able to produce nodal directions that are generally consistent with the *a priori* biological expectations in the particular context of aggregation, any resulting *a posteriori* findings based on NPA calculations need to be investigated in light of both the biological content of the network models and the specific biology induced in the underlying experiment, as we are advocating in general for network scoring [1, 16]. For example, here we noted that TNF-mediated activation of MAPKs led to a large impact on the "Replicative Senescence" network through a minority of network nodes in a single region of the network. Given the fact that this region contained a causal inconsistency that was resolved by the SST algorithm, additional focus can be given to investigating the findings to ensure that they are biologically relevant. Even with these caveats, our overall investigation of network nodal directions and scores broadly confirmed the pertinence of the SST algorithm in the concrete cases of NPA and BIF calculations.

The above application of the SST algorithm in the biological context of a concrete data set suggests a final remark. Taking Eq. (3) from the previous section and replacing the exhaustive summation over all *N*(*G*) spanning trees of the graph *G* by the sampled subset  obtained with Aldous’ method yields the following expression for the aggregated quantity *X*_*G*_:
7

where *X*(*t*) corresponds to the aggregation of the nodal values *X*_*n*_ over the spanning tree *t*. By considering the "sampled" distribution of the aggregated scores , it is possible to extract quantities such as the "most representative spanning tree" t* for which |*X*(*t*) - *X*_*G*_| is minimal. Because a spanning tree *t* unambiguously defines a balanced subgraph of *G*, we can define G* as the subgraph of *G* corresponding to t*. The fact that t* and G* depend on the nodal values *X*_*n*_ implies that they can potentially change when considering several comparisons in NPA and BIF calculations, as in the example presented above. This can lead to valuable data-driven edge-level biological insights that can be explored in future applications of the SST algorithm, such as for instance in the case of time-course or dose-dependent expression data.

## Conclusions

In this note, we have described the SST algorithm, which uses random walks for aggregating nodal values over arbitrary signed graphs, including large "causally inconsistent" network models. We used a remarkable property of suitably generated random walks, which provide a representative sampling among all the spanning trees of the graph and an approximation of the nodal effective weights as the average over the sampled spanning trees. We applied the SST algorithm to several biological causal networks where the pertinence of its results could be confirmed using biologically quasi-equivalent but graph-theoretically simpler networks. This SST algorithm is applicable in a variety of situations requiring the aggregation of nodal values (e.g., gene differential expression and nodal NPA scores [16, 19]) over a signed graph and is scalable to arbitrary graph sizes.

## Appendix

### (1) Concepts from systems biology

#### Reverse causal reasoning

In recent years, (reverse) causal reasoning has become increasingly popular in systems biology [1, 9, 17, 20, 21]. It is based on the idea that the differential expressions measured in transcriptomics are consequences of the changes in the activities of upstream controlling entities (such as, but not limited to, transcription factor proteins). It represents an alternative to the often implicit assumption that transcript differential expressions reflect changes in the activity of the corresponding proteins, which is used when integrating transcriptomics data and pathways and networks [2].

#### Causal network models

Reverse causal reasoning is based on high-quality literature-derived prior biological knowledge organized in causal network models. In this study, we used the same naming convention as in our previous publications [1]: a "causal network model" is a set of edges representing experimentally supported causal relationships "→" and "—|" between changes affecting the corresponding nodes, which consist of various biological entities (excluding transcript differential expressions) encoded in BEL [13]. An example of a "causal network model" is the "Cell Cycle" network [6]. A "causal network model augmented with downstream measurables" is a "causal network model" that additionally contains all the causal edges ending in nodes containing transcript differential expressions (termed "RNA abundance" in BEL). In the literature, the two cases are not always distinguished [9, 20, 21].

#### Causal (in)consistency

A causal network model is said to be causally consistent if the relative sign in {-1,+1} between any pair of nodes {***A***, ***B***} of the network is unambiguous [1]. The relative sign between nodes ***A*** and ***B*** is given by the product of the edge signs in {-1,+1} along any (non-oriented) path relating the two nodes ***A*** and ***B***. If there exists at least one node pair in the network for which the relative sign is ambiguous and thus dependent on the chosen path, then the network is said to be causally inconsistent. The causal (in)consistency is a fundamental property of causal network models and is analogous to the notion of balance in graph theory (see below). Alternative equivalent formulations of this property are possible, in particular based on Harary’s theorem [22].

#### Reference node of a causal network model

As a causal network model only contains the relative signs in {-1,+1} of its nodes, it needs to be assigned a reference node that fixes the "absolute" sign at the network level. The reference node can be any node in the network whose level of activity is positively related to the activity of the network as a whole (see the section "Constructing a HYP from a causal network model" in [1]). For instance, the node "cell proliferation" is the reference node for the causal network model "Cell Cycle" [6], as increases (or decreases) in cell proliferation and the cell cycle are always closed related. Reference nodes have been assigned to all the published causal network models [16]. Note that the positive sign given *a priori* to the reference node within a network model is not related to the sign of the change of biological activity it underwent in the *a posteriori* context of an experiment.

#### Network perturbation amplitudes

The idea of "network perturbation amplitudes" (NPA) extends previous concepts, such as "signaling pathway impact" analysis (SPIA), where real-values scores have been calculated to quantify the changes in the biological activity at pathway or network levels [8]. "Real-valued scores" are used in contrast with the strictly positive enrichment p-values that do not contain information about the sign of the activity change and therefore cannot distinguish activation from inhibition. Compared with SPIA, one of the novelties of the NPA approach is the use of reverse causal reasoning to calculate the intermediate node-level scores [1].

### (2) Concepts from graph theory

#### Graphs

Graphs are the mathematical objects underlying the network models. They are defined as *G* = (Nodes, Edges) where "Nodes" is a set of nodes and "Edges" is a set of edges relating pairs of nodes. Causal network models result in "oriented signed graphs": the edges have an orientation corresponding to the causal direction between the start and the end nodes (not relevant in this study), as well as a sign from {-1,+1} describing the relative sign between the changes in the nodes, with +1 = "→" = "an increase (decrease) in the start node causes an increase (decrease) in the end node", and -1 = "—|" = "an increase (decrease) in the start node cause a decrease (increase) in the end node". As mentioned above, the causal (in)consistency property of network models is equivalent to the notion of balance of signed graphs, which has been studied in graph theory for several decades [22, 23].

It is worth remembering that signed graphs are of interest in a variety of other contexts besides the mathematical description of causal network models considered here. They are quite extensively used in the modeling of regulation and signaling in systems biology, not to mention the quantitative analysis of social networks [24], which includes social psychology, where they initially appeared [23]. This observation suggests that the SST algorithm described in this note may be of interest in these contexts also, because it does not depend on the specific type of real-valued quantities that are aggregated over the signed graph.

#### Spanning trees

A spanning tree *t* of a connected graph *G* is a connected subgraph of *G* that includes all the nodes of *G* and the minimal number of edges to remain connected [14]. By construction, any spanning tree *t* is balanced. Therefore, a spanning tree *t* extracted from an unbalanced graph *G* differs from *G* by at least *z* edges, where *z* is the line index of balance of *G* [23].

### (3) Implementation of the SST algorithm for BEL-encoded causal network models

The description of the SST algorithm given in the "Findings" section implicitly assumed that all the edges of the graph can be treated equally. For semantic reasons, this assumption does not hold in the case of causal network models encoded in BEL [13]. Intramolecular edges relating a protein and its activity carry a higher relevance, and thus a higher likelihood of being retained in the spanning tree than direct intermolecular edges relating the activities of two causally-linked but different proteins that are known to directly interact. Indirect intermolecular edges relating the activities of two causally linked proteins that are not known to interact directly carry a lower weight. Edges leading to changes in gene expression carry the lowest weight because they tend to occur over longer timescales than the other edges in the network and are often related to feedback loops. Therefore we slightly adapted Step 2 of the SST algorithm to account for this extra granularity in the edges of the network models encoded in BEL:

For each node, a walker randomly chooses an edge to traverse, according to probabilities given for each edge. The relative probabilities are fixed as:

- 1 for intramolecular edges (relationships between molecules and their activities),
- 1/2 for direct intermolecular edges (direct bindings and increases/decreases),
- 1/3 for indirect intermolecular edges (increases/decreases),
- 1/4 for expression edges (relationships leading to changes in RNA abundance).

This semi-quantitative modeling approach adequately implements the differences among the edges of the BEL-encoded network models while guaranteeing a stable execution of the SST algorithm, by not too strongly affecting the originally uniform spanning tree sampling procedure. Besides its use in the applications of the SST algorithm reported in this note, this modeling scheme has been also satisfactorily employed in two other studies [16, 19]. Some of the SST-based NPA scores were compared with the corresponding phenotypic endpoints, which were separately measured (for instance NPA scores for the "Cell Cycle" network model and fractions of cells in S-phase measured by flow cytometry [25]). Both gave consistent results that confirmed the modeling approach introduced here.

### (4) The TNF treatment dataset

This data set is publicly available under the ArrayExpress identifier E-MTAB-1027. The data processing used to obtain the 16 treatment vs. control comparisons is described in the paragraph "Data processing and algorithm implementation" of our previous publication [1]. The NPA GPI scores for these 16 comparisons were calculated for both the causally inconsistent and the pruned causally consistent versions of the eight relevant network models. This yielded 16 pairs of values for each network, which were globally compared using Pearson correlation coefficients.

## Electronic supplementary material

Additional file 1: Table S1: Causally inconsistent biological networks used for the evaluation of the SST algorithm. The networks from the Cell Proliferation (green, [6]), cellular stress (pink, [10]), DNA damage/autophagy/cell death/senescence (DACS, yellow, [11]), and pulmonary inflammation (blue, [12]) publications are indicated together with the edges that were removed to obtain the pruned causally consistent versions. The biological motivations for removal are briefly explained in the "Rationale" column. Note that "catof" represents the catalytic activity of a protein, "kaof" represents the kinase activity of a protein, "taof" represents the transcriptional activity of a protein, and "exp" represents the expression of a gene. The corresponding articles provide more details on the actual network content. (PDF 85 KB)

## Abbreviations

BEL: Biological expression language
BIF: Biological impact factor
GPI: Geometric perturbation index
HYP: Hypothesis subnetwork
IFFL: Incoherent feed-forward loop
NHBE: Normal human bronchial epithelial
NPA: Network perturbation amplitude
ROC: Receiver operating characteristic
SPIA: Signaling pathway impact analysis
SST: Sampling spanning trees.
