# Supplementary material for: An algorithm for score aggregation over causal biological networks based on random walk sampling
Source: BMC Res Notes. 2014 Aug 11;7:516. doi: 10.1186/1756-0500-7-516 (PMC4266947; doi:10.1186/1756-0500-7-516)
Supplement: Supplementary file 1 — Additional file 1: Table S1: Causally inconsistent biological networks used for the evaluation of the SST algorithm. The networks from the Cell Proliferation (green, [6]), cellular stress (pink, [10]), DNA damage/autophagy/cell death/senescence (DACS, yellow, [11]), and pulmonary inflammation (blue, [12]) publications are indicated together with the edges that were removed to obtain the pruned causally consistent versions. The biological motivations for removal are briefly explained in the "Rationale" column. Note that "catof" represents the catalytic activity of a protein, "kaof" represents the kinase activity of a protein, "taof" represents the transcriptional activity of a protein, and "exp" represents the expression of a gene. The corresponding articles provide more details on the actual network content. (PDF 85 KB) [file 13104_2014_3074_MOESM1_ESM.pdf]

Additional File

Table S1. Causally inconsistent biological networks used for the evaluation of the SST algorithm. The networks from the Cell Proliferation (green, [6]), cellular stress (pink, [10]), DNA damage/autophagy/cell death/senescence (DACS, yellow,[11]), and pulmonary inflammation (blue, [12]) publications are indicated together with the edges that were removed to obtain the pruned causally consistent versions. The biological motivations for removal are briefly explained in the "Rationale" column. Note that "catof" represents the catalytic activity of a protein, "kaof" represents the kinase activity of a protein, "taof" represents the transcriptional activity of a protein, and "exp" represents the expression of a gene. The corresponding articles provide more details on the actual network content.

Network model	Edges to be removed	Rationale
Hedgehog	exp(HHIP) → HHIP<br>exp(PTCH1) → PTCH1	HHIP and PTCH1 are negative regulators of proliferation that are induced by GLI2, a positive regulator of proliferation. Removals ensure that HHIP, PTCH1, and GLI1 maintain their expected relationships with proliferation.
Notch	exp(HES1) → HES1	HES1, a negative regulator of proliferation, is induced by Notch proteins, which are positive regulators of proliferation. Removal ensures the expected relationships with proliferation are maintained.
Nuclear Receptors	taof(NR4A1) —| cell proliferation	While NR4A1 has been reported to be both a positive and negative regulator of proliferation, it is canonically a positive regulator so the negative edge is removed.
PGE2	catof(PTGES)/ catof(PTGES2)/ catof(PTGES3) —| Prostaglandin H2<br>catof(PTGER3) —| 3'-5'-Cyclic AMP	PTGES family converts Prostaglandin H2 to Prostaglandin E2, so inhibitory relationship is artifact of representation of the reaction. Removals ensure PGE2, PGH2, and PTGES family activity are all positively related.<br>PTGER3is negative regulator of cAMP, but other PTGER family members are positive regulators. Removal ensures PTGER family members all negatively related to proliferation.
Hypoxic Stress	exp(TXNIP) → TXNIP<br>Response to Hypoxia —| exp(KDR)<br>Response to Hypoxia —|/→ Reactive Oxygen Species<br>taof(EPAS1) → exp(CFTR)	TXNIP is a negative regulator of HIF1A whose expression is HIF1A-mediated. Removal ensures TXNIP has negative relationship with HIF1A.<br>Response to hypoxia inhibition of KDR is inconsistent with direct evidence of KDR regulation by HIF1A and EPAS1.<br>Relationship between Response to Hypoxia regulation and ROS is ambiguous, and likely not causal. Edge removal ensures that more causal edges determine their relationship.<br>EPAS1 activation of CFTP expression is inconsistent with negative regulation of CFTR by HIF1A and CFTR's role in Cl^- regulation in response to hypoxia
Osmotic Stress	Hyperoxia —| catof(sodium/potassium-exchanging ATPase complex)	Edge is not supported by direct mechanistic knowledge, whereas other edges in causal inconsistent loop are.
DNA Damage-<br>Components affecting<br>TP63 activity	All edges connected to WWP1 and catof(WWP1)	WWP1 has an ambiguous relationship with respect to apoptosis, and thus is not uniquely indicative of increased or decreased apoptosis.
Replicative Senescence	paof(DUSP4) to replicative cell aging	Edge is not supported by direct mechanistic knowledge, whereas other edges in causal inconsistent loop are.

Dendritic Cell Activation	taof(STAT3) —| taof(NFKB Complex Hs)	TLR9 is a positive regulator of dendritic cell activation through canonical TLR to NFKP pathway, and a negative regulator through STAT3 activation. More mechanistic evidence for canonical pathway, so STAT3 to NFKB edge removed.
Macrophage Activation	taof(AP-1 Complex Hs) → exp(Il10)<br>CD44 —| taof(NFKB Complex Hs)	AP-1 mediated IL10 expression is opposed by NFKB-mediated suppression of IL10 expression. To preserve IL10's role as anti-inflammatory, positive connection from AP-1 to IL10 is removed. CD44 can inhibit NFKB, but generally is pro-inflammatory so edge was removed to maintain its pro-inflammatory role.
Macrophage Differentiation	exp(VDR) → VDR	This edge is part of a feedback loop where mature macrophages repress expression of VDR. This network is meant to capture the mechanisms of macrophage differentiation, so removal of this edge results in VDR being positively related to macrophage differentiation as expected.
Mast Cell Activation	kaof(PRKCD) —| mast cell activation	Ca^{2+} is a positive regulator of mast cell activation by inducing histyamine release, and is a negative regulator of mast cell activation through activation of protein kinase C family members. Choice to remove this edge in the Ca^{2+} dependent inhibition of mast cell activation was arbitrary.
Megakaryocyte Differentiation	taof(GATA1) —| taof(STAT3)	While GATA1 has been shown to directly inhibit STAT3, both transcription factors are positive regulators of megakaryocyte differentiation, and edge removal ensure this relationship is maintained for both proteins.
NK Cell Activation	IL4 → kaof(PI3K Family Hs)<br>TGFB1 —| KLRK1	IL4 has an overall negative relationship with NK cell activation, while PI3K has an overall positive relationship. IL4 activation of PI3K edge was removed to ensure expected overall relationships were maintained.<br>TGFB1 has been shown to decrease levels of KLRK1 and increase the activity of PI3K, both of which are positive regulators of NK cell activation. TGFB1 regulation of PI3K is more canonical, so edge between TGFB1 and KLRK1 was removed.
Neutrophil Chemotaxis	ELA2 —| catof(MMP12)<br>IL8 → Acute Phase Protein Family Hs<br>All edges involving SERPINA1:IL8<br>catof(SOD2) —| H2O2	ELA2 has overall positive effect on neutrophil chemotaxis, so edge in specific route of negative regulation of chemotaxis through MMP12 and IL8 is removed to maintain this expected overall relationship.<br>IL8 can lead to increased levels of acute phase protein family members, at least one of which (SPRPINEA1) can bind and inhibit IL8. Edge removal ensures overall positive relationship between IL8 and neutrophil chemotaxis.<br>SERPINEA1 can increase neutrophil chemotaxis through inhibition of ELA2, and inhibit neutrophil chemotaxis through inhibition of IL8. Arbitrary choice to treat SERPINE 1A only as an activator in chemotaxis is enabled by removal of all edges involving SERPINEA1/IL8 complex.<br>While SOD2 generally decreases reactive oxygen species (ROS), it has also been shown to increase intracellular H2O2. Edge removal ensures overall negative relationship between SOD2 and ROS, as well as with neutrophil chemotaxis.
Neutrophil Response	kaof(JNK family Hs) —| taof(NFKB Complex Hs)	JNK family kinases are known to inhibit NFKB activity, but both proteins are known activators of neutrophil response. Edge removal ensures positive relationship with neutrophil chemotaxis
Tc Response	exp(BCL2) → BCL2	IL15 can lead to the negative regulation of T-cell activation through IL2RB, and positive regulation though increased expression of BCL2. Edge removal ensures an overall positive relationship for BCL2, and negative relationship for IL15.
Th1 Differentiation	DNMT3A—| IFNG	DNMT3A levels are negatively related to the levels of IL4, a negative regulator of Th1 cell differentiation, and negatively related to the levels of IFNG, which is expressed in Th1 cells. Edge removal ensures that network focuses on events leading to Th1 cell differentiation rather than the state after differentiation has occurred.
Th17 Differentiation	exp(SOCS3) → SOCS3	STAT3, a positive regulator of Th17 cell differentiation, causes expression of its own inhibitor SOCS3. Edge removal ensures that STAT3 maintains a positive relationship with differentiation, and SOCS3, via inhibition of STAT3, maintains a negative relationship with differentiation.
